# New insights and advances of sodium-glucose cotransporter 2 inhibitors in heart failure

**DOI:** 10.3389/fcvm.2022.903902

**Published:** 2022-09-15

**Authors:** Juexing Li, Lei Zhou, Hui Gong

**Affiliations:** ^1^Department of Cardiology, Jinshan Hospital of Fudan University, Shanghai, China; ^2^Department of Internal Medicine, Shanghai Medical College, Fudan University, Shanghai, China

**Keywords:** heart failure, mechanisms, metabolism, sodium-glucose cotransporter 2 inhibitors (SGLT2is), diabetes

## Abstract

Sodium-glucose cotransporter 2 inhibitors (SGLT2is) are newly emerging insulin-independent anti-hyperglycemic agents that work independently of β-cells. Quite a few large-scale clinical trials have proven the cardiovascular protective function of SGLT2is in both diabetic and non-diabetic patients. By searching all relevant terms related to our topics over the previous 3 years, including all the names of agents and their brands in PubMed, here we review the mechanisms underlying the improvement of heart failure. We also discuss the interaction of various mechanisms proposed by diverse works of literature, including corresponding and opposing viewpoints to support each subtopic. The regulation of diuresis, sodium excretion, weight loss, better blood pressure control, stimulation of hematocrit and erythropoietin, metabolism remodeling, protection from structural dysregulation, and other potential mechanisms of SGLT2i contributing to heart failure improvement have all been discussed in this manuscript. Although some remain debatable or even contradictory, those newly emerging agents hold great promise for the future in cardiology-related therapies, and more research needs to be conducted to confirm their functionality, particularly in metabolism, Na^+^-H^+^ exchange protein, and myeloid angiogenic cells.

## Introduction

Heart failure, a chronic pathological condition, is a universal prevailing disease, with more than 26 million sufferers (1). It has come to light that several factors are attributed to the heart failure process, including disorders of the structure, function, rhythm, and conductive systems (2). Such abnormal diseases place a tremendous cost on society and the economy. Thus, we should give sufficient emphasis to seeking more economical and effective treatment techniques for heart failure.

SGLT2is, a new class of oral anti-hyperglycemic agents, were required by the U.S. FDA to undergo cardiovascular safety testing prior to being officially marketed. The outcomes exceed expectations—SGLT2is have shown unexpected cardiorenal protection in both heart failure with reduced ejection fraction (HFrEF) and heart failure with preserved ejection fraction (HFpEF) in several clinical trials (3–7), which have been detailed in Table 1. As a result, SGLT2is are recommended as one of the fundamental treatments for heart failure in recent guidelines, particularly the 2022 AHA/ACC/HFSA one (8). Together with ACEI/ARNI, BB, and MRA, they are collectively called “new quadruple therapy.” It is noteworthy that SGLT2is can be applied across the entire stages of heart failure management, even if in the at-risk stage for heart failure.

**Table 1:** Contribution of SGLT2i to heart failure in major clinical trials.

	**EMPA-REG (3)**	**CANVAS (4)**	**DECLARE-TIMI (5)**	**DAPA-HF (6)**	**CREDENCE (193)**	**EMPEROR-reduced (197)**	**RECEDE-CHF (198)**	**REFORM (199, 200)**	**EMPEROR-preserved (7)**	**Empire-HF (201)**	**PRESERVED-HF (202)**	**SOLOIST-WHF (203)**
Year of publication	2015	2017	2019	2019	2019	2020	2020	2020	2021	2021	2021	2021
Region	42 countries	30 countries	33 countries	20 countries	34 countries	20 countries	UK	UK	23 countries	Denmark	USA	32 countries
Characteristics of population	T2DM	T2DM	T2DM	HFrEF with and without T2DM (NYHA class II-IV)	T2DM with kidney disease	HFrEF with and without T2DM (NYHA class II-IV)	HFrEF with T2DM	HFrEF with T2DM (NYHA class I-III)	HFpEF with and without T2DM (NYHA class II-IV)	HFrEF, with and without T2DM (NYHA class I-III)	HFpEF with T2DM or pre-diabetes (NYHA class II-IV)	T2DM with recent worsening HF
Drugs	Empagliflozin	Canagliflozin	Dapagliflozin	Dapagliflozin	Canagliflozin	Empagliflozin	Empagliflozin	Dapagliflozin	Empagliflozin	Empagliflozin	Dapagliflozin	Sotagliflozin (SGLT1/2i)
Number of participants, n	7,020	10,142	17,160	4,744	4,401	3,730	23	56	5,988	391	324	1,222
Median follow-up, years	3.1	3.6	4.2	1.5	2.62	1.34	0.15	1	2.18	2	0.23	0.75
Mean age, years old	63	63.3	63.9	66.2	63.0	67.2	69.8	67.1	71.8	68	70.0	70.0
Gender, male%	71	64.2	63.1	76.2	66.1	76.5	73.9	66.1	55.4	78	43	66.3
Mean BMI, kg/m^2^	30.6	32.0	32.1	28.2	31.3	28.0	33.9	32.5	29.77	29	34.7	30.4
HbA1c, %	8.06	8.3	8.3	N/A	8.3	N/A	7.9	7.72	N/A	5.8	6.1	7.1
CVD risk factor, %	99	65.6	40.5	100	50.4	100	100	100	100	100	100	100
Prior HF, %	10	13.9	9.9	100	14.8	100	100	100	100	100	100	100
3P-MACE^*^	0.86 (0.74–0.99)	0.86 (0.75–0.97)	0.93 (0.84–1.03)	N/A	0.80 (0.67–0.95)	N/A	N/A	N/A	N/A	N/A	N/A	0.72 (0.56–0.92)
CV death^*^	0.62 (0.49–0.77)	0.87 (0.72–1.06)	0.98 (0.82–1.17)	0.82 (0.69–0.98)	0.78 (0.61-−1.00)	0.92 (0.75–1.12)	N/A	N/A	0.91 (0.76–1.09)	N/A	N/A	0.84 (0.58–1.22)
Non-fatal myocardial infarction^*^	0.87 (0.70–1.09)	0.85 (0.69–1.05)	0.89 (0.77–1.01)	N/A	N/A	N/A	N/A	N/A	N/A	N/A	N/A	N/A
Non-fatal stroke^*^	1.24 (0.92–1.67)	0.90 (0.71–1.15)	0.73 (0.61–0.88)	N/A	N/A	N/A	N/A	N/A	N/A	N/A	N/A	N/A
CV death or HHF^*^	0.66 (0.55–0.79)	0.78 (0.67–0.91)	0.83 (0.73–0.95)	0.75 (0.65–0.85)	0.74 (0.63–0.86)	0.75 (0.65–0.86)	N/A	N/A	0.79 (0.69–0.90)	N/A	N/A	0.67 (0.52–0.85)
All-cause mortality^*^	0.68 (0.57–0.82)	0.87 (0.74–1.01)	0.93 (0.82–1.04)	0.83 (0.71–0.97)	0.83 (0.68–1.02)	0.92 (0.77–1.10)	N/A	N/A	1.00 (0.87–1.15)	N/A	N/A	0.82 (0.59–1.14)
HHF^*^	0.65 (0.50–0.85)	0.67 (0.52–0.87)	0.73 (0.61–0.88)	0.70 (0.59–0.83)	0.61 (0.47–0.80)	0.69 (0.59–0.81)	N/A	N/A	0.71 (0.60–0.83)	N/A	N/A	0.64 (0.49–0.83)
HbA1c changes, %^*^	N/A	−0.58 (−0.61 to 0.56),	−0.42 (0.40–0.45)	−0.24 (−0.34 to −0.13)	−0.31 (0.26–0.37)	−0.16 (−0.25 to −0.08)	N/A	−1.49 (−6.95 to 3.97)	N/A	−3.9 (−6.8 to −1.1)	N/A	N/A
Serum creatinine changes, mg/dL^*^	N/A	N/A	N/A	0.02 (0.01–0.03)	N/A	N/A	−1.66 (−3.07 to −0.25)	1.46 (−5.56–8.47)	N/A	2.1 (−2.3–6.4)	N/A	N/A
Hemoglobin changes, g/dL^*^	N/A	N/A	N/A	N/A	N/A	N/A	N/A	0.86 (0.27–1.46)	N/A	0.4 (0.2–0.5)	N/A	N/A
Hematocrit changes, %^*^	N/A	N/A	N/A	2.41 (2.21–2.62)	N/A	2.36 (2.08–2.63)	0.018 (−0.05 to 0.042)	2.89 (1.14–4.64)	N/A	0.02 (0.01–0.03)	N/A	N/A
NT-proBNP changes, pg/ml^*^	N/A	N/A	N/A	−303 (−457 to −150)	N/A	0.87 (0.82–0.93)	283.4 (−835.8–1,402.3)	N/A	N/A	N/A	0.99 (0.88–1.12)	N/A
Weight/ BMI changes (kg, m/kg^2^)^*^	N/A	−1.60 (−1.70 to −1.51)	−1.8 (1.7–2.0)	−0.87 (−1.11 to −0.62)	−0.80 (0.69–0.92)	−0.82 (1.18–0.45)	−1.71 (−2.90 to −0.53)	N/A	N/A	−1.4 (−2.3 to −0·6)	−0.72 (−1.42 to −0.01)	N/A
Systolic blood pressure changes, mmHg^*^	N/A	−3.93 (−4.30–3.56)	−2.7 (2.4–3.0)	−1.27 (−2.09 to −0.45)	−3.30 (2.73–3.87)	−0.7 (−1.8 to 0.4)	−6.8 (−17.6 to 4.0)	−4.7 (−14.51–5.11)	N/A	−5.4 (−9.3 to −1.6)	−0.6 (−4.4 to 3.3)	N/A

* Hazard Ratio or Absolute difference (95% CI).

BMI, body mass index; HbA1c, hemoglobin A1c; CVD, cardiovascular diseases; HF, heart failure; 3P-MACE, cardiovascular death, non-fatal myocardial infarction, and non-fatal stroke; HHF, hospitalization for heart failure; NT-proBNP, N-terminal pro–B-type natriuretic peptide.

Those clinical trial results are groundbreaking. Thus, researchers have conducted quite a few studies concerning the cardioprotective function of SGLT2is. It has been thoroughly researched in the regulation of diuresis, sodium excretion, weight loss, blood pressure improvement, hematocrit and erythropoietin stimulation, metabolism remolding, protection from structural dysregulation, etc. Some of them have been sophisticatedly proven, but the in-depth mechanisms and some other recently emerging mechanism theories such as Na^+^-H^+^ exchange protein and myeloid angiogenic cells remain vague.

Thus, this manuscript reviews these newly emerging studies from the perspectives of natriuresis, weight loss, blood pressure reduction, etc., containing all the research that may be concerned with this topic during the past 3 years so as to identify the potential mechanisms and provide a full landscape of its benefits to patients with heart failure.

## The anti-hyperglycemic mechanisms of SGLT2i

The maximum transport capacity (T_max_) of the proximal tubule in humans is 500 g of glucose per day under normal conditions. Thus, as blood circulates through, almost all glucose will be reabsorbed. When blood glucose levels are above a certain threshold, which implies they are higher than the renal tubules' capacity to reabsorb glucose, glucose starts to show up in the urine and is then expelled (9, 10).

**Figure 1:**
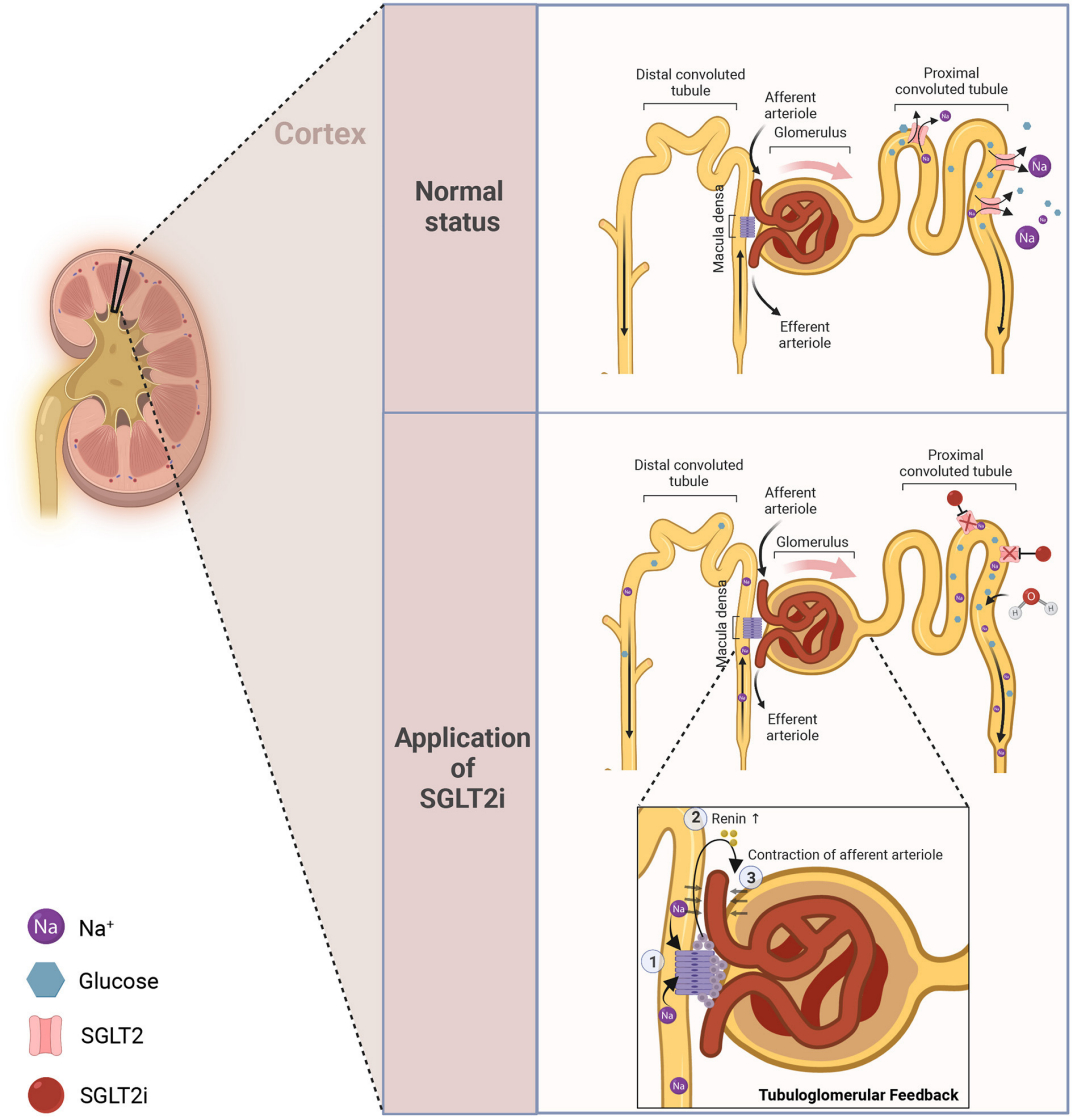
The anti-hyperglycemic mechanism of SGLT2i. In normal status, glucose and Na^+^ can be efficiently reabsorbed at the site of the proximal convoluted tubule to maintain glucose homeostasis. Once applied with SGLT2i, such reabsorption will be inhibited, thereby leading to diuresis and natriuresis.

The active transporters or symporters (SGLTs) and the facilitated transporters or uniporters (GLUTs) are two different types of glucose transporters that are known to contribute to glucose homeostasis (11). The SGLT family contributes to the active absorption of glucose/galactose in the site of the intestine as well as the reabsorption of glucose in the kidney (12). SGLT receptors are categorized into six types in human bodies (11). SGLT1/2 are mainly enriched in the kidney, while SGLT2, a symporter for Na^+^ and glucose, plays a considerable role during glucose reabsorption in the kidney (11). The new anti-hyperglycemic drug SGLT2i directly binds to the corresponding receptor to curb the reabsorption of glucose at the site of the proximal convoluted tubule, and the unabsorbed part is excreted from the body, thereby achieving the effect of blood sugar lowering (Figure 1, which was created with BioRender.com).

## Underlying mechanisms of SGLT2i's benefits to patients with heart failure

### Diuresis and natriuresis

The course and prognosis of heart failure are closely tied to sodium and water retention, which is responsible for 90% of heart failure hospitalizations (13). Water and sodium retention result from reduced circulation, decreased renal blood flow, and elevated aldosterone when the heart's pumping capacity is compromised (14). Furthermore, the blood detained in vein vessels will seep into the interstitial fluid under the pressure of capillaries, and those penetrative fluids remain there when the volume of the fluids exceeds the surrounding cells' abilities of absorption (remarkably, the fluid in lower limbs) (15).

In terms of its mode of action, SGLT2i is distinct from traditional diuretics. With the application of SGLT2i, glucose reabsorption is hindered at the site of renal tubules, and therefore the unabsorbed portion will flow into the distal nephron. However, water can still be reabsorbed unimpededly as usual during this period. Thus, the reduction of the osmotic gradient leads to a decrease in water reabsorption, termed osmotic diuresis (16). One of the critical factors in a significantly reduced rate of heart failure deterioration is a decrease in circulation volume, which is caused by the natriuretic action of SGLT2i. But the eyes-catching role of diuresis in the process of heart failure has been undermined by another large clinical trial which concludes that patients with a high propensity for fluid retention show no remarkably extra benefits compared to the control group under the application of empagliflozin (17). Beyond this diuresis function, an additional independent element during the progression of heart failure may be the improvement of pulmonary artery diastolic pressure (18).

On the other hand, prior research has shown that diabetes patients have higher sodium levels in their skin and muscles (19). Moreover, tissue sodium is closely linked to ventricular hypertrophy regardless of blood pressure and water-sodium retention conditions. It is reported that the application of dapagliflozin for 6 weeks can decrease tissue sodium in patients with T2DM (20) compared to control groups, thereby reducing the risk of left ventricular hypertrophy and chronic heart failure. In contrast, in an emergency scenario, increased glycosuria rather than natriuresis, with SGLT2i utilization, contributes to improving the patient's state (21).

### Reduction of body weight and fat content

Excessive adipose tissue exerts vital functions in the onset and progression of heart failure (22). Several studies have revealed that SGLT2i plays a crucial part in weight shedding during its pharmacological action in both types of diabetes (23–25), and the baseline body mass index will not impair the effectiveness of SGLT2i, contrary to what is known as the “obesity paradox” (26).

Attributed to the calories loss caused by the excretion of glucose (27) and enhanced hypothalamic insulin responsiveness (28), the application of SGLT2i can significantly reduce the weight of patients, leading to the reduction of total fat mass, subdermal fat, visceral fat, and liver fat content (29–33). What's more, it has been revealed that SGLT2i can reduce the size of adipocytes in the perivascular adipose tissue (34) and be capable of inducing mitochondrial biogenesis through the AMPK/SIRT1 pathway and β3-adrenoceptor-cAMP-PKA signaling pathway, thereby increasing the energy consumption of adipocytes directly *in vivo* (35–37). Some researchers believe that it is possible to achieve pounds shedding by inducing the beigeing of fat (38), while beigeing refers to a specific metabolism remolding of fat tissue (39). Additionally, a recent study reveals that the activated innervation of intra-adipose sympathetic induced by SGLT2i in mice with a high-calorie diet also contributes to such an increased energy consumption (37, 40). Fibroblast growth factor 21 (FGF21) is a coordinator for the SGLT2i-dependent decline in adiposity and the trigger of lipolysis during weight loss (41). This mediator can perform a role in the activation of the nervous system, thereby achieving the biological effect of white/brown adipose tissue and the induction of thermogenesis (40, 42).

**Figure 2:**
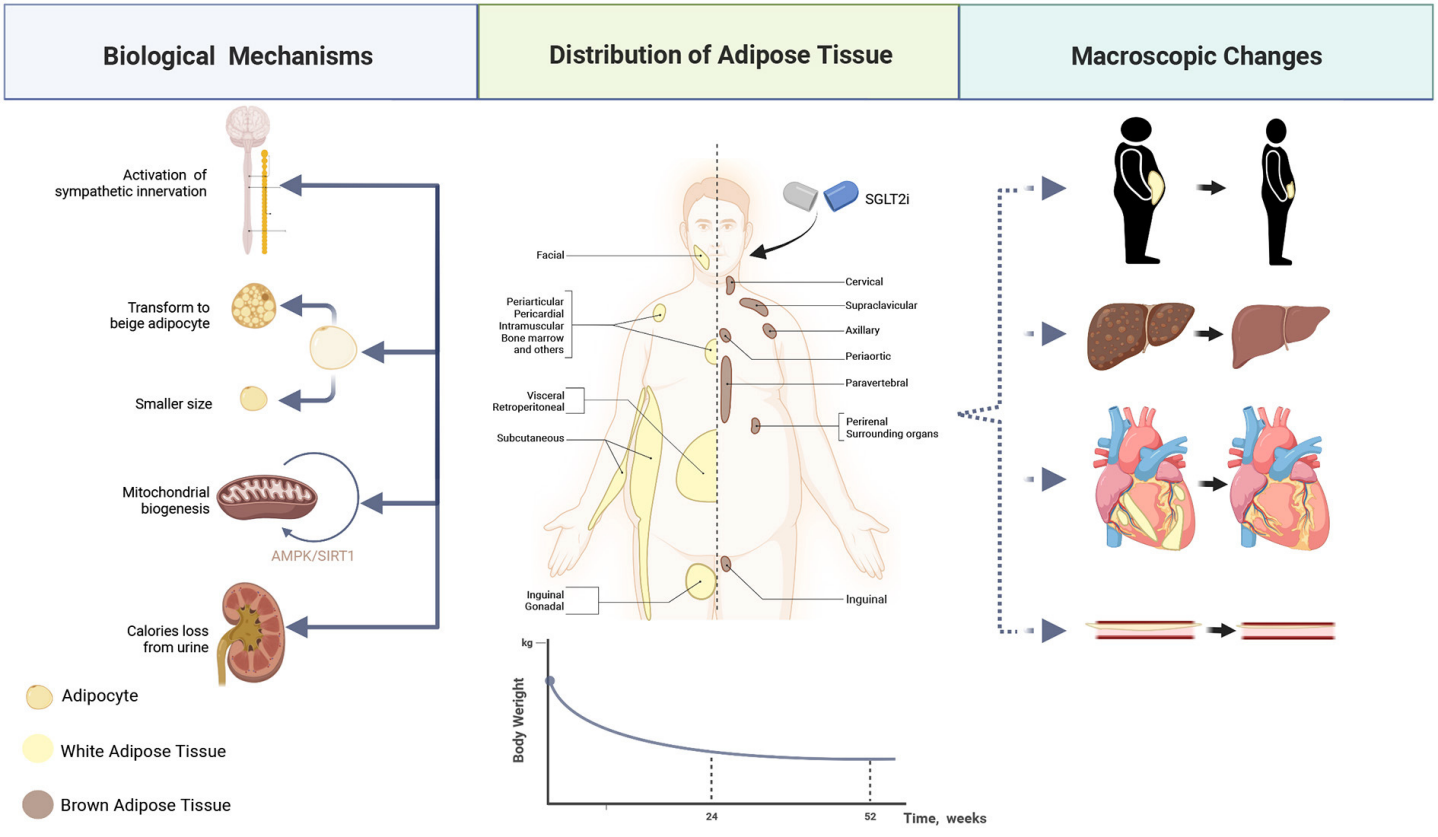
Mechanisms of weight loss. Full view of how SGLT2i affects adipocytes—how to reduce the size, and how to stimulate its transformation to beige adipocytes, as well as how it makes the body pounds shed directly *via* calories loss. Consequently, the subcutaneous, visceral, and perivascular fat content decreases under the treatment of SGLT2i.

This weight loss impact does not last indefinitely; it progressively reaches a plateau between 24 and 52 weeks. These phenomena may be explained by the fact that adipose tissue has an anti-lipolytic function, and the decline in leptin levels has boosted compensatory eating (43–45). Such increased intake is a higher calorie intake (46) with a constant proportion of nutrients as previously, although those intake calories are ultimately countered by glucose loss (28, 47). Figure 2, which was produced using BioRender.com, shows intricate mechanisms. Therefore, although SGLT2i cannot permanently treat obesity, lowering body weight and fat percentage may still positively affect the cardiovascular system.

### Improvement of blood pressure

The blood volume in the body is tightly connected to blood pressure levels. Nevertheless, it has been observed that volume changes and urinary sodium excretion, under the application of SGLT2i, do not significantly lower blood pressure (48), suggesting that additional processes may have been at work throughout this blood pressure-lowering process.

In clinical trials, the 24 h ambulatory blood pressure shows a slight reduction among people suffering from nocturnal hypertension, diabetes, or salt sensitivity with the application of SGLT2i, thereby lowering the incidence of heart failure and the tolls of cardiovascular mortality (49). Such an efficacy may be achieved by regulating renal HIF-1α, impairing inflammation and oxidative stress (50), regulating the function of the paraventricular nucleus of the hypothalamus (PVN) (51), and sGC pathways (52). A meta-analysis of 43 RCT studies reveals that under SGLT2i treatment, patients' systolic blood pressure (SBP) decreases by a mean of 2.46 mmHg and diastolic blood pressure (DBP) by a mean of 1.46 mmHg (25, 53), while another meta-analysis confirms that SGLT2i significantly reduced 24 h dynamic SBP and dynamic DBP, and such an effect is concluded as SGLT2i-like effect (54). Therefore, SGLT2i generates cardiovascular benefits by reducing blood volume, lowering blood pressure, and improving ventricular load through its diuretic and natriuretic effects. In the meantime, it also exerts cardiovascular effects by regulating the central nervous system and affecting PVN.

### Promotion of hematocrit and erythropoietin

An endogenous glycoprotein hormone called erythropoietin (EPO) can promote erythropoiesis (More details on this concept have been fully explicated in the reviews by Lappin, T.R. and Koury, M.J.) (55, 56). EPO is mostly produced by the kidney and in minor quantities by the liver. At the same time, a hypoxic environment will trigger its production. Moreover, the values of hematocrit and hemoglobin within normal ranges are negatively correlated to the incidence or prognosis of cardiovascular diseases (57–60). EPO and hematocrit significantly rise after using empagliflozin for 1–6 months, causing a continuous rise in blood cells that peaks in 2–3 months (29, 61, 62), and this tendency is independent of the initial levels of anemia (63).

These changes in EPO and hematocrit can be elaborated as follows: (1) Decreased plasma volume and blood concentration induced by osmotic diuresis (64), but some hold the contradictory view (2) Increased erythropoiesis and hematocrit *via* inhibiting hepcidin and regulating other iron regulatory proteins (65), (3) The application of SGLT2i can reduce the reabsorption of glucose and reduce the expensed ATP in Na^+^/K^+^ pumps, thereby decreasing the metabolic load of the EPO-related cells and improving the status of hypoxia. To a certain extent, such an improvement of hypoxia restores myofibroblasts back to erythropoietin-producing fibroblasts, leading to enhanced hematopoietic function and increased hematocrit (64, 66, 67), (4) Inhibition of SGLT2 will induce the activity of HIF and SIRT1. The HIF-2α activation in the kidney and liver can promote the production of erythropoietin (68) and strengthen the activation of SIRT1, resulting in modification of macrophage polarization which promotes anti-inflammatory phenotypes and impairs myocardial inflammation (69). (5) *In vivo*, it has also been observed that dapagliflozin can increase endogenous antioxidant enzymes and regulate fibrosis markers in renal tissues, thereby reducing the oxidative stress of rat kidneys.

### Improvement of cardiac energy metabolism

Incessant contraction of the heart requires enough energy to support. Previous studies (70) have concluded that when the heart goes through the process of failing, abnormalities in cardiac energy metabolism, particularly in advanced stages, will occur, often manifested as increased glucose intake (mainly on anaerobic glycolysis) (71–73), reduced fatty acid oxidation, and decreased ketone oxidation, coincident with severely impaired mitochondrial function, leading to an overall depleting ATP content (More on the metabolic profile of the failing heart can be found in the reviews by Stanley et al. (70). Thus, it is essential to shift the substrate preference to satisfy such a pathological energy demand. However, such an alternation comes with a price—patients may be trapped in a vicious circle that worsens their more advanced heart failure.

The chief role SGLT2i plays in the metabolism of a failing heart is to improve the overall cardiac ATP production through the increased consumption of fatty acids, ketone bodies, and amino acids, with a concomitant decrease in glucose utilization (73, 74). Quantitatively, it can increase the overall ATP content by ~30% (75). Concerning glucose metabolism, SGLT2i-treated heart failure models show a reduced intake of cardiac glucose and a decreased level of metabolism-related enzymes, mainly shifting the substrate preference to ketone bodies, free fatty acids (73). Also, SGLT2i improves glucose tolerance and insulin resistance *via* several mechanisms such as inflammasome suppression (76), while the plasma insulin has been down-regulated and the glucagon shows a rise (37, 41, 77).

In mitochondrial dysfunction models, the improved potency of fatty acid oxidation, the reduced aggregation of fatty acid intermediates, as well as the improved synthetic efficiency of mitochondrial bioenergy are attributed to the function of SGLT2i, thereby enhancing the oxidation of the fatty acids in cardiac mitochondria and, as a result, avoiding mitochondrial failure (36, 78–80). A similar trend has also been observed in clinical trials (74). From another perspective, both *in vitro* and *in vivo*, SGLT2i has been confirmed to act as an activator of the AMPK signaling pathway to regulate metabolism, ameliorate inflammation, and maintain mitochondrial homeostasis (81–83).

SGLT2i can upregulate the plasma ketone levels, in part, mediated by the reduced plasma glucose levels and decreased insulin levels (41), coincident with a climb in free fatty acids (84, 85). As a result of the ongoing disorder of glucose, the failing heart becomes more dependent on ketone bodies to serve as extra fuel (86, 87). It will simulate a pseudo fasting state and give priority to the utilization of ketone bodies (29, 80, 88, 89). Such an effect of ketone bodies production is directly induced without other factors' mediation (90). SGLT2i also upregulates ketogenic enzymes and transporters in livers, kidneys, and intestines, which, accordingly, increases the level of beta-hydroxybutyric acid in the bodies and tissues (91). However, this promising hypothesis remains debatable and controversial due to the absence of further cogent and robust evidence (92). Some find that the efficiency of ketone bodies oxidation remains approximately unchanged under the application of SGLT2i and believe that the increased production of ATP benefits, in part, from the extra supplement of ketone bodies (75). Nevertheless, some researchers observe the contradictory results that bioavailability and oxidation have been increased by SGLT2i (93). This vague issue requires more research to tackle with. And such an increase in ketone bodies may, in turn, bring about worries about diabetic ketoacidosis, which is worthy of caution during its application.

### Alleviation of inflammation

As both a cause and a result of cardiovascular diseases, inflammatory cytokines, such as interleukin-1 (IL-1), interleukin-6 (IL-6), tumor necrosis factor (TNF), galectin-3, and others, have a very significant positive link with the pathophysiology and development of these conditions (94). The inflammatory profile of heart failure has been well-illustrated in the reviews by Murphy et al. (95) and Adamo et al. (96). Inflammasomes were initially reported as a kind of complex for caspase-activator (97). Moreover, now, they are referred to as multiprotein signaling complexes, including NOD-like receptor 1 (NLRP1), NOD-like receptor 3 (NLRP3), NOD-like receptor 6 (NLRP6), absent in melanoma 2 (AIM2), etc., which regulate inflammatory processes as well as anti-pathogen defenses (98, 99), inducing an increase in pro-inflammatory cytokines (100, 101). Those factors have a close connection with impaired blood vessels (102), disordered mitochondrial function (103), elevated oxidative stress, deteriorated hypertension (104), progressed atherosclerosis (99, 105), and advanced myocardial damage (106), etc.

Inflammatory cytokines and inflammasomes can both be suppressed by SGLT2i. To be more specific, it has been confirmed in rodent models that the crucial inflammasome NLRP3, a complex of caspase-1, IL-1β, and IL-18 cytokine-triggering factors (76, 101, 107), can be suppressed under the application of SGLT2i (108–110), which is, in part, calcium-mediated (105, 109). Several studies have shown that the AMPK pathway can restrain the increase of inflammasomes (111), and treatment with SGLT2i can bring about a climb of AMPK phosphorylation in LPS-treated cardio-fibroblasts (69), thereby avoiding the consequently augmented inflammation.

From another perspective, β-hydroxybutyrate also plays a significant role in regulating inflammasomes and inflammatory cytokines. Oscillated levels of β-hydroxybutyrate and insulin go hand in hand with inflammation (or inflammasome) levels. It has been established that SGLT2i can increase the β-hydroxybutyrate levels (discussed in the previous section), and this climb acts as a defender against endoplasmic reticulum stress-related inflammasomes *via* the activation of AMPK (111). However, some researchers believe this regulation is not AMPK-dependent. They reveal that the suppressive function of β-hydroxybutyrate on inflammasomes has nothing to do with fasting-related mechanisms such as AMPK, ROS, or other factors. Instead, it works by reducing the apoptosis-associated speck-like protein containing CARD (ASC) oligomerization and speck formation (112, 113), and this can be mediated through the G-protein-coupled receptor 109a (Gpr109a)–NLRP3 pathway associated with the increased influx of extracellular calcium (105). Furthermore, β-hydroxybutyrate is able to regulate mitochondrial protein acetylation, lowering the levels of NLRP3 and inflammatory cytokines, thus achieving the benefits in HFpEF models (103).

The most logical explanation for how SGLT2i reduces inflammation seems to be that inflammation is closely associated with hyperglycemia (114–117), and these negative effects will be lessened as soon as the glucose level falls. Recently, more attention has been paid to the regulation of insulin, which is regarded universally as an anti-inflammatory factor in most cases (118–120). However, recent studies report that in some specific circumstances, insulin can trigger the pro-inflammatory pattern of macrophages, thereby contributing to a climb in IL-β mediated by activated inflammasome and overproduction of ROS (121, 122). Such a function of insulin has been validated in a clinical setting by other research conducted by La Grotta et al. (123). Likewise, given the glucose-lowering function of SGLT2i, the insulin shows a decrease along with the dropped levels of blood glucose correspondingly, contributing to the avoidance of progressed inflammation and increased inflammasome. Moreover, alleviated insulin resistance, which has been mentioned above, also brings benefits to inflammation reduction, because the insulin resistance status possesses strong links with pro-inflammatory and inflammatory states (124–126). So, insulin-related mechanisms can at least partially mediate SGLT2i's ability to suppress inflammation.

### Reduction of oxidative stress

Oxidative stress is a biological process characterized by high levels of free radicals that is accompanied by detrimental consequences (127, 128). It is well-known that a high level of blood glucose can induce oxidative stress intracellularly (129, 130), and such a stress-related function is negatively correlated with the level of HbA1c (131). SGLT2i can significantly contribute to the reduction of oxidative stress through a variety of mechanisms besides blood sugar lowering in its capacity as an anti-hyperglycemic drug.

Both *in vivo* and *in vitro*, it is reported that SGLT2i can attenuate cell apoptosis induced by endoplasmic reticulum stress (132–136). This process may be mediated by the curbed TGF-β/Smad pathway (137, 138) and the activated Nrf2/ARE signaling (139). Furthermore, SGLT2i use can eliminate intracellular ROS, block ROS-activated signaling pathways, and improve the hyperglycemic state in this process as well (140, 141). It can also normalize the size and quantity of mitochondria by regulating autophagy and reducing mitochondrial abnormalities attributed to myocardial infarction (142). What's more, SGLT2i is able to suppress inflammatory factors and oxidative stress by inhibiting the NO-sGC-cGMP pathway and curbing the polymerization of PKGIa (141, 143). Therefore, the methods above, as well as SGLT2i-mediated suppression of endoplasmic reticulum stress-induced apoptosis, can support cardioprotective function in heart failure.

### Reduced sympathetic hyperexcitability

In patients with T2DM, hyperglycemia and hyperinsulinemia often lead to sympathetic hyperexcitation, which consequently results in aggravated hypertension and an increased occurrence of other cardiovascular diseases (144–146). Contrary to our expectations, the heart rate, under the effects of those mechanisms above, including diuresis, decreased blood volume, and decreased blood pressure, has not shown a significant increase, suggesting that SGLT2i can reduce the heart rate of patients with a heart rate faster than 70 bpm (147). It may have the potential effect of inhibiting sympathetic nerve excitation. This role can be described as a “mediator” of SGLT2i to produce cardiovascular benefits.

*In vivo*, dapagliflozin significantly improves mice's blood pressure and endothelial function *via* reduced excitability of the sympathetic nervous system and down-regulated IL-6 (148). Interestingly, such a function goes hand in hand with the natriuretic effect of SGLT2i rather than its sugar-lowering function (149). At the same time, it is well-known that SGLT2 receptors are mainly enriched in the tissues of the central nervous system involved in autonomous regulation, and SGLT2i may affect the central nervous system in an uncharted way, exerting a sympathetic inhibition effect (51). In clinical trials of those who suffer from acute myocardial infarction with T2DM, the application of SGLT2i for 24 weeks improves sympathetic hyperexcitability and parasympathetic nerve function through changes in hemodynamics, myocardial energy metabolism, and function of the hepatic vagus nerve (150). On the other hand, from the perspective of cardiorenal syndrome, SGLT2i can also indirectly suppress sympathetic hyperexcitability by improving renal function.

However, there are limited clinical studies on how SGLT2i affects the sympathetic nervous system in the human body, and further clinical studies are required to be conducted.

### Improvement of left ventricular remodeling and myocardial necrosis

It has been reported that SGLT2i can prevent and delay heart remodeling (135, 151–153), thereby protecting against the deterioration of heart failure.

In various animal models, SGLT2i shows the ability to prevent atrial remodeling, electrical remodeling (154, 155), and endothelial dysfunction (156–158). SGLT2i is confirmed to be capable of delaying the progression of left ventricular concentric hypertrophy in aorta cells by reducing sympathetic nerve tension and inflammation in the aorta (159). At the same time, the left ventricular systolic and diastolic functions have been enhanced, and the stiffness of cardiomyocytes has been improved (160, 161). Additionally, SGLT2i directly affects the phenotype and function of human cardiac myofibroblasts by weakening their activities and regulating cell-mediated collagen remodeling. Also, it can regulate the PERK-eIF2α-CHOP axis and activate the SIRT1 pathway (135, 162), thereby improving cardiac remodeling and heart failure.

On the other hand, SGLT2i can also exert a cardioprotective effect on patients suffering from myocardial infarction, preventing heart failure after that occurrence. It can reduce the infarct area (163, 164) independently of the blood sugar state, improving the heart function, remodeling, and metabolic state after myocardial infarction, etc. (93, 165, 166). However, cardiomyocytes chiefly express SGLT1 and seldom express SGLT2. It has been reported that specific knockdown of SGLT1 can reduce the infarct size in mice through function with EGFR (167), and SGLT1 knockdown can also ameliorate cardiac fibrosis and pyroptosis *in vivo* (168, 169), suggesting the potential ability of SGLT1 to provide cardiovascular benefits. So, is there a possibility that SGLT1/2i will have more beneficial impacts compared with SGLT2i? The SOLOIST-WHF trial shows that SGLT1/2 brings great benefits to the patients (which has also been detailed in Table 1), but whether SGLT1/2i is superior to SGLT2i or not remains unclear. Moreover, what is worth mentioning is that dual inhibitors of SGLT1/2 have been reported to exacerbate cardiac dysfunction after myocardial infarction in rats (170), which contradicts the assumption and clinical trials. The underlying reason for such a phenomenon requires additional preclinical research.

### Inhibition of Na^+^-H^+^ exchange protein

It has been revealed that empagliflozin can curb the activity of NHE1 (Na^+^/H^+^ Exchange Protein 1) in the hearts of mice, rats, and rabbits, thereby reducing the concentration of sodium and calcium in cardiomyocytes (171–173). Also, it can function as a direct cardiac effect to inhibit the NHE of the isolated intact heart and delay the occurrence of ischemic contracture of the heart in the absence of insulin (174). Similarly, empagliflozin can reduce the calcium sensitivity of human myocardial myofilament (175), improving the diastolic dysfunction of the human myocardium and inhibiting NHE in human bodies. By and large, SGLT2i successfully reduces the amount of Na^+^ entering cells by inhibiting NHE1 and improves the contractile dysfunction in heart failure by normalizing the intracellular pH, thereby producing cardiovascular benefits. However, there are some opposite voices that the activity of cardiac NHE1 will not be influenced by empagliflozin or other SGLT2i, as under the application of therapeutic doses, empagliflozin shows no regulatory effect on the concentration of Na^+^ (176). They believe that the role of SGLT2i in heart failure should not be interpreted as being mediated by myocardial NHE1 or intracellular Na^+^. In general, the function of SGLT2i for NHE is controversial since the clinical research data is very scarce, and more evidence is still required.

### Regulation of adipokines and epicardial adipose tissue

Adipose tissues secrete a large amount of biologically active substances collectively known as adipokines, which have been fully described in the reviews by Lelis et al. (177) and Kim et al. (178). Adipokines are in a harmonious balance—adiponectin and SFPR5 can down-regulate the expression of many pro-inflammatory mediators and prevent a variety of obesity-related endocrine or cardiovascular diseases (179, 180). Simultaneously, leptin can stimulate inflammatory responses and up-regulate pro-inflammatory elements such as TNF- and IL-6, hastening the onset and progression of cardiovascular disease (181, 182). Leptin and adiponectin exert contrary effects on subclinical inflammation and insulin resistance. Under the pathological condition of obesity, hypertrophic lipocytes and immunocytes in adipose tissue will activate and accelerate the chronic pro-inflammatory response and regulate the secretion of adipokines and other regulatory factors, with a deterioration of cardiometabolic diseases (183). Meta-analysis shows that SGLT2i can effectively reduce circulating leptin levels, increase circulating adiponectin levels (184), and inhibit the excretion of IL-6 and TNF-α in animal aortas (137, 159, 185), thereby producing cardiac vascular protection and delaying the progression of heart failure. Furthermore, SGLT2i can also reduce the size of epicardial adipose tissue (186) in patients by improving systemic micro-inflammation (187) and alleviating adipose-related vascular diseases (188), benefiting heart failure patients.

### Improved level of myeloid angiogenic cells level

Myeloid angiogenic cells are abbreviated to MAC, which means vascular endothelial progenitor cells, and play a vital role in the pathological process of atherosclerosis. Its quantity is negatively correlated with cardiovascular events, serving as a biomarker (189). The regulation of SGLT2i on such progenitor cells expression is controversial, and some studies confirm that treatment with SGLT2i can up-regulate the expression of vascular progenitor cells (190). However, some studies believe that SGLT2i does not increase circulating stem cells and endothelial progenitor cells, while such an increase of endothelial progenitor cells may be indirectly achieved through the improvement of blood glucose level, and SGLT2i has no direct effect on endothelial progenitor cells (191, 192). In conclusion, the relationship between SGLT2i and endothelial progenitor cells is an emerging direction, and the results of existing studies are inconsistent and controversial.

## Renal benefits and its resultant heart failure benefits

With several clinical trials finishing in succession, it has been approved that SGLT2i will bring quite a few renal benefits (3–5, 193, 194), and those renal benefits will, synchronously, favor the treatment of heart failure. The dominant mechanism for its renal-protection is attributed to the tubuloglomerular feedback change under the application of SGLT2i (which has also been shown in Figure 1). Briefly speaking, when applied with SGLT2i, the reabsorption of Na^+^ and glucose will be impaired. Then, the macula dense can detect such changes, activating the tubuloglomerular feedback and inducing the contraction of afferent arteriole to maintain homeostasis. As a result, some pathological states, such as high perfusion, high load, and high filtration of the glomerulus, will be relieved, leading to sound renal protection (195, 196). From another perspective, it has been discussed in previous sections that SGLT2i can also be capable of reducing the kidney's metabolic burden and mitigating oxidative stress or inflammation at that site *in vivo*. The improved renal function will, in turn, bring benefits to heart failure *via* increased EPO generation, etc., thereby creating a virtuous cycle for cardiorenal interaction.

## Conclusion

**Figure 3:**
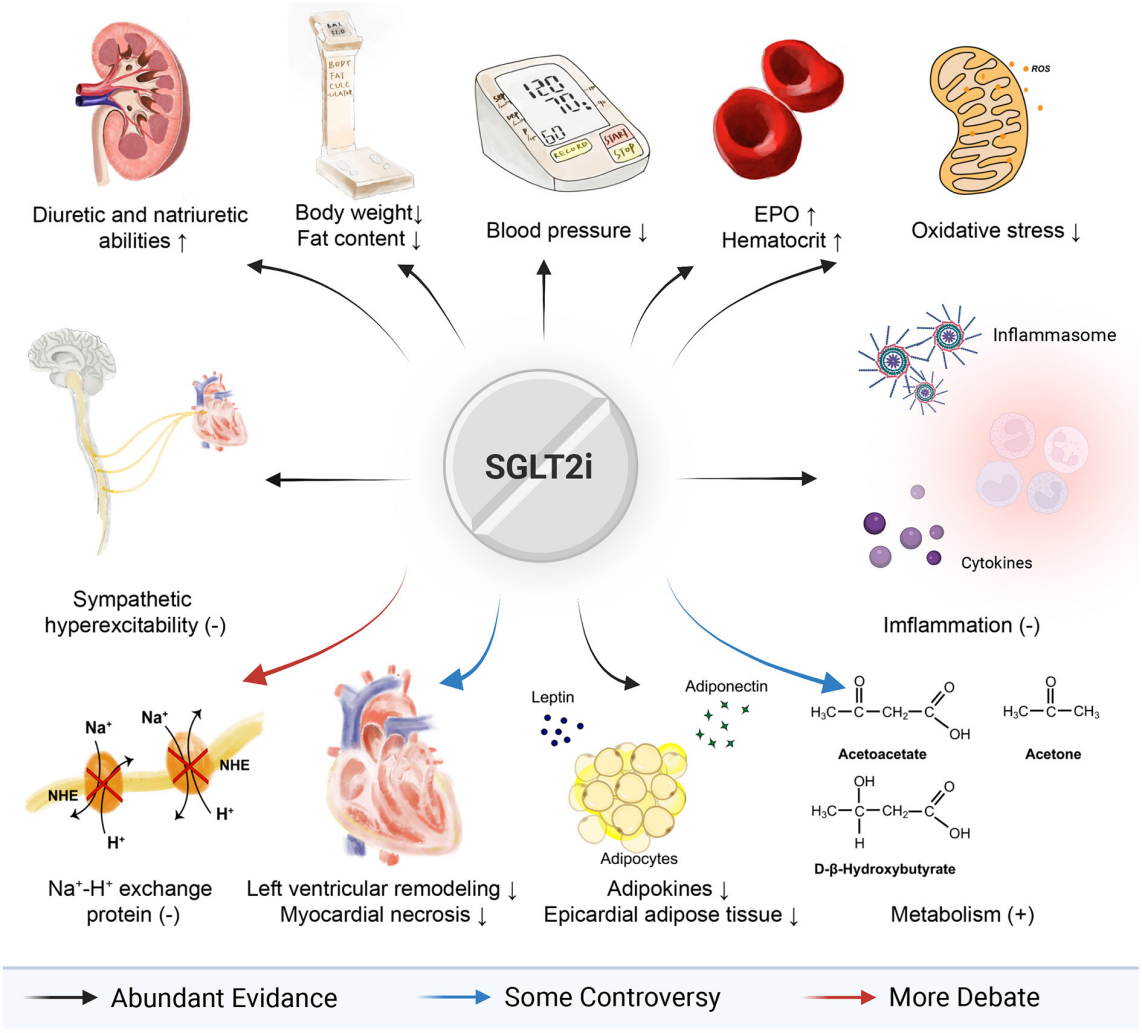
Full view of SGLT2i in heart failure.

With the advent of SGLT2i, a breakthrough in the treatment of cardiovascular diseases has appeared. At this stage, SGLT2i has been applied clinically and has been accepted in a variety of guidelines. By and large, SGLT2i achieves its cardiovascular outcomes and benefits patients with heart failure through combined effects of multiple ways (which has been visualized in Figure 3), which means it is hard to fully explain its rapid and comprehensive benefits only by one of those mechanisms. Nevertheless, some of those mechanisms mentioned above are still uncertain, while others are contradictory and controversial, like Na^+^-H^+^ exchange protein and myeloid angiogenic cells. Thus, further studies on cells, animals, and clinics ought to be supplemented. It is promising that in-depth research will further open up more paths and hold great promise for cardiovascular diseases, including heart failure, myocardial hypertrophy, and even treatments for comorbidity.

## Author contributions

JL was responsible for paper collecting and article writing. LZ was responsible for the revision of this article. HG took responsibility of ideas generation and suggestion making. All authors contributed to the article and approved the submitted version.

## Funding

This work was supported by grants from the Natural Science Foundation of Shanghai (No. 19JC1415703).

## Conflict of interest

The authors declare that the research was conducted in the absence of any commercial or financial relationships that could be construed as a potential conflict of interest.

## Publisher's note

All claims expressed in this article are solely those of the authors and do not necessarily represent those of their affiliated organizations, or those of the publisher, the editors and the reviewers. Any product that may be evaluated in this article, or claim that may be made by its manufacturer, is not guaranteed or endorsed by the publisher.
